# Phenolic Compounds from Five Ericaceae Species Leaves and Their Related Bioavailability and Health Benefits

**DOI:** 10.3390/molecules24112046

**Published:** 2019-05-29

**Authors:** Bianca Eugenia Ștefănescu, Katalin Szabo, Andrei Mocan, Gianina Crişan

**Affiliations:** 1 Department of Pharmaceutical Botany, “Iuliu Hațieganu” University of Medicine and Pharmacy, 23, Ghe. Marinescu Street, 400337 Cluj-Napoca, Romania; stefanescu.bianca@umfcluj.ro (B.E.Ș.); mocan.andrei@umfcluj.ro (A.M.); 2 Institute of Life Sciences, University of Agricultural Sciences and Veterinary Medicine, Cluj-Napoca, CaleaMănăştur 3-5, 400372 Cluj-Napoca, Romania; 3 Laboratory of Chromatography, Institute of Advanced Horticulture Research of Transylvania, University of Agricultural Sciences and Veterinary Medicine, 400372 Cluj-Napoca, Romania

**Keywords:** Ericaceae, *Vaccinium* spp. leaves, phenolic components, arbutin, chlorogenic acid, quercetin

## Abstract

Some species of the Ericaceae family have been intensively studied because of the beneficial health impact, known since ancient times, of their chemical components. Since most studies focus on the effects of fruit consumption, this review aims to highlight the phenolic components present in the leaves. For this purpose, five species from Ericaceae family (bilberry—*Vaccinium myrtillus* L., lingonberry—*V. vitis-idaea* L., bog bilberry—*V. uliginosum* L., blueberry—*V. corymbosum* L. and bearberry—*Arctostapylos uva-ursi* L.) were considered, four of which can be found in spontaneous flora. The chemical composition of the leaves revealed three major phenolic compounds: chlorogenic acid, quercetin and arbutin. The health promoting functions of these compounds, such as antioxidant and anti-inflammatory properties that could have preventive effects for cardiovascular disease, neurodegenerative disorders, cancer, and obesity, have been exemplified by both in vitro and in vivo studies in this review. Furthermore, the importance of bioaccessibility and bioavailability of the phenolic compounds have been summarized. The findings highlight the fact that leaves of some Ericaceae species deserve increased attention and should be studied more profoundly for their biological activities, especially those from spontaneous flora.

## 1. Introduction

The Ericaceae is a large family of angiosperm plants that includes herbs, dwarf shrubs, shrubs and trees which are found most commonly in acid and infertile growing conditions. There are approximately 4250 known species in this family distributed in 124 genera and 9 subfamilies [1]. The subfamily of Vaccinioidae includes numerous species of berries with health promoting properties like cranberry, blueberry, bilberry, bog bilberry, lingonberry, huckleberry and others. The health benefits of these fruits are attributed to antioxidants which are highly effective in the control of free radical production; they prevent the undesirable effects of reactive oxygen species, and support the antioxidant and detoxifying mechanisms in the organism [2]. 

Phenolic compounds of plant origin and their antioxidant properties are an intensively studied topic and increasing evidence proves their protective activity against a multitude of non-communicable human conditions [3,4]. Polyphenols are organic chemical compounds from the phenol group, containing minimum two hydroxyl groups attached to the aromatic ring. They occur naturally in plants like grapes, green tea, olives, blueberries, nuts, cocoa, yerba mate and others, with highest concentrations in the fruit’s skins; many of them show a strong antioxidant effect [5,6,7].

*Vaccinium* spp. are a good source of natural phenolic compounds, e.g., anthocyanins, proanthocyanidins, quercetin and hydroxycinnamic acids and many others [8,9,10]. Among them anthocyanins provide the natural pigmentation of the berries (red, violet, purple, and blue colors) and exhibit therapeutic benefits including the integrity of genomic DNA, cardio- and neuroprotective effects, as well as anti-inflammatory, and anti-carcinogenic properties [11]; quercetin exerts health beneficial effects in preventing a number of chronic diseases, including cardiovascular and neurodegenerative diseases, through modulating the signaling pathways and gene expression involved in these processes [12].

Due to the high content of phenolic compounds, Ericaceae species are used for therapeutic purposes [13] and many of these can be found in the Vaccinioidae subfamily. Most of the scientific evidence regarding the health benefits of *Vacccinium* species relates to the berry fruits consumption and a smaller percent highlights the proficiency of bioactive compounds found in the leaves. While the *Vaccinium* fruits have a seasonal nature that implies high harvesting and storage costs, the leaves are available in most seasons and some even in wintertime (e.g., lingonberry and bear berry). This aspect is important because the phenolic content of the berries is sensitive to processing and storage conditions, with some of the compounds decreasing considerably over time [14].

Three major *Vaccinium* fruit crops (blueberry, cranberry, and lingonberry) have been domesticated in the twentieth century due to their benefic effects on human health, however there is some evidence that polyphenol, anthocyanin, flavonoid contents and antioxidant capacity is higher in wild blueberry varieties compared to cultivated ones [15]. Bilberry and the fruits of a number of other non-cultivated *Vaccinium* species also show great potential as new crops [16].

This paper aims at reviewing the content and properties of the phenolic compounds present in the leaves of five widely used Ericaceae species: bilberry, lingonberry, bog bilberry, blueberry and bearberry along with their bioavailability in the human organism and their health benefits.

## 2. Phenolic Compounds Present in *Vaccinium* Species Leaves

Phenolic compounds are a large group of phytochemicals which represent secondary plant metabolites. Some of the most usual phenolic compounds are simple phenolic acids and flavonoids; they altogether occur as soluble conjugated or insoluble forms [17]. Phenolic acids and phenolic alcohols are molecules with only one phenol ring; the other phenolic compounds contain more than one phenol ring and a diversity of molecules, all having a polyphenol structure. Polyphenols are classified into multiple groups according to the number of phenol rings present in their structure and to the binding elements that bond these rings together. The representative groups of polyphenols are: flavonoids, lignans, phenolic acids, stilbenes and tannins [18]. The in vivo antioxidant activities of phenolic compounds were comprehensively reviewed and the concluding remark was that more clinical trials are needed to develop future and effective alternatives in order to improve the health of individuals; moreover, the interactions between variables like bioavailability and bio-efficacy, nutrient-phytochemicals and drug-phytochemicals should be evaluated for a more complete image of the phenolic compounds [2].

### 2.1. Bilberry Leaves

Bilberry (*Vaccinium myrtillus* L.), also known as European blueberry or whortleberry, is a perennial, wild and small deciduous shrub, which grows in the mountains and forests of Europe. Abundant scientific evidence shows that the representatives of all groups of phenolic compounds are present in the fruits and leaves of bilberry [10,19,20,21]. A special feature of these species is the high content of anthocyanins. Probably flavonoids in the leaves are a mechanism of defense against stress caused by excess light, since the flavonoid metabolism is activated by ultraviolet light. Furthermore, the phenolic content in proanthocyanidins, kaempferol, quercetin and hydroxycinnamic acids is higher in the leaves compared to the fruits [21]. In autumn, bilberry leaves change color from green to red, this phenomena having a great impact on the phenolic composition and implicit in their properties.

#### 2.1.1. Phenolic Content of the Bilberry Leaves

A study conducted on organ-specific distribution of phenolic compounds revealed that red bilberry leaves contain anthocyanins, in concentrations of 882 μg/g, while the green leaves did not contain anthocyanins [21]. Other phenolic compounds like quercetin, kaempferol, hydroxycinnamic acids are in higher percentages in the red leaves compared to the green ones (Table 1). Moreover, the leaves of bilberry, (both green and red) represent an important source for procyanidins which have proven anti-radical activity [21,22,23].

The phenolic profile of the leaves was progressively framed by Fraisse et al., who reported that chlorogenic acid and quercetin-3-*O*-glucuronide are the main compounds [22], statement confirmed by Oszmianski et al., who identified and quantified three phenolic compounds in bilberry leaves: chlorogenic acid,3-*O*-p-coumaroylquinic acid and quercetin-3-*O*-glucuronide [24]. Furthermore, Hokkanen et al. detected 35 phenolic compounds in the methanolic extracts of bilberry leaves using LC/TOF-MS and LC/MS/MS [25]. Among the 35 compounds found in bilberry leaves, the main compounds were: quercetin-3-*O*-glucuronide, *trans*-chlorogenic acid, *cis*-chlorogenic acid and cinchonain Ia, Ib, Ic or Id with 30%, 17%, 11% and 6% shares, respectively, of the combined LC/TOF-MS peak areas of all detected phenolic compounds. Along with the main compounds, several phenolic acids were also quantified, like caffeoylshikimic acid, feroylquinic acid isomer, coumaroylquinic acid isomer and traces of caffeic acid and *p*-coumaric acid, as well as flavonols like quercetin-3-*O*-β-galactoside, quercetin-3-*O*-(4″-HMG)-α-rhamnoside, quercetin-3-*O*-arabinoside, quercetin-3-*O*-glucoside, quercetin-3-*O*-α-rhamnoside (quercitrin), quercetin, and three kaempferol glycosides. The authors also reported other bioactive phenolic compounds like flavan-3-ols, three proanthocyanidin trimers, type A/B 1, type A/B 2 and type B and two coumaroyl iridoids, revealing the most complex phenolic profile of bilberry leaves [25]. 

Martz et al. conducted two studies to investigate the phenolic composition (soluble phenolics) of bilberry leaves [26]. In the first study the phenolic compounds from bilberry leaves were examined during their foliar development. The leaves were collected from three different sites of the spontaneous flora: a clear-cut area, an old spruce forest and a higher altitude fell. Phenolic compounds were extracted with acidified methanol-water mixtures. The main compounds in all samples were chlorogenic acid derivatives, representing 55.5% of the total soluble phenolics, followed by flavonol glycosides with 29.6%, mainly quercetin derivatives and also myricetin derivatives and kaempferol-glucuronide. Catechins, hydroxycinnamic acids, and proanthocyanidins were detected in every sample, but in lower percentages: 7.4%, 6.3% and 1.1%, respectively. In each growing site, the levels of the total phenolics were similar in immature leaves, however at the ending of the foliar development the phenolic content was significantly lower in the samples from the forest compared to the other two sites. These results are in agreement with a previous study, confirming that the phenolic composition is higher in leaves exposed directly to sunlight compared to those in the shade [23].

The second study investigated phenolics from bilberry leaves along environmental gradients; the leaves were collected from 116 study sites of the Finland National Forest Inventory network, situated at different altitudes and latitudes [26]. The results showed that leaves from higher latitudes and higher altitudes had greater soluble phenolic and flavonol content, higher antioxidant capacity, and lower chlorogenic acid derivatives content. Difference in phenolic composition occurs depending on the leaves colors as well; a complex study analyzed red and green bilberry leaves and identified 21 phenolic compounds in the red leaves and 18 in the green leaves [27]. The phenolic composition was comparable for both type of leaves, although anthocyanins like cyanidin-3-*O*-galactoside, cyanidin-3-*O*-glucoside and cyanidin-3-*O*-arabinoside were identified only in the red bilberry leaves. Some of the detected compounds were reported for the first time, like: B-type procyanidin dimer-hexoside, caffeoyl-glucoside derivative and two derivatives of *p*-coumaroyl-glucoside. The most abundant groups of phenolic compounds were phenolic acids (chlorogenic acids, caffeoyl derivative and *p*-coumaroyl derivatives) and flavonols (quercetin and kaempferol derivatives flavanols and procyanidins, like catechin, epicatechin and A and B type procyanidins dimer and trimer were identified as well. The study highlights that the red leaves have higher content of chlorogenic acids and flavonol glycosides than the green leaves [27]. 

Recently, Bujor et al. studied the phenolic composition of the bilberry leaves collected in May, July and September and identified 62 phenolic compounds [28]. The phenolic composition was comparatively similar for the three harvesting periods. The authors identified two types of hydroxycinnamic acid derivatives: caffeic acid derivatives and *p*-coumaric acid derivatives. Flavonol glycosides and flavanols were also present in the leaves. Caffeic acid derivatives were found as esters of caffeic acid with quinic acid, shikimic acid, monotropein or a hexose moiety and were in higher concentration compared to coumaric acid derivatives (Table 2). 

The caffeic acid derivatives identified in leaves were: 5-O-caffeoylquinic acid, 5-O-caffeoylquinic acid hexosides, caffeoylquinic acid derivatives, caffeoyl malonylhexosides, caffeoyl monotropein and caffeoylshikimic acid. From the group of coumaric acid derivatives compounds like *p*-coumaroylquinic acid, hexosides of *p*-coumaric acid, *p*-coumaroyl diacetylhexosides, *p*-coumaroyl triacetylhexosides, *p*-coumaroyl malonylhexoside, *p*-coumaroyl malonyldihexoside and *p*-coumaroyl monotropein or dihydromonotropein derivatives have been identified. In addition, hydroxycinnamic acid derivatives flavonol glycosides like one kaempferol hexuronide and some quercetin glycosides were also present, and flavanols, like epicathecin and oligomeric flavanols, the latter containing a wide variety of B-type dimers, trimers and tetramers. The bilberry leaves analyzed in this study also revealed some isomeric cinchonains I and cinchonains II content, bringing to light a complex phytochemical profile (supplementary material 1).

#### 2.1.2. Therapeutic Uses and Biological Properties of the Bilberry Leaves

Bilberry leaves are traditionally and currently used for treating the affections of the urinary tract through their astringent and antiseptic properties. Various types of extracts like infusions or decoctions have anti-bacterial, anti-inflammatory and hypoglycemic activities as well, due to the presence of phenolic compounds [29,30].

The anti-bacterial property of bilberry leaves extracts was validated by several studies; the anti-staphylococcal activity against two strains of *Staphylococcus aureus, ATCC 29213* and *H9*, resulted in a minimum inhibitory concentration (MIC) of 1500 μg/mL and a minimum bactericidal concentration (MBC) of 750 μg/mL [31]. The synergistic anti-staphylococcal effect of the extracts with antibiotics was also tested in the study; the MIC of some commonly used antibiotics against staphylococcal infections (linezolid and vancomycin), in combination with bilberry extract was decreased from 4 μg/mL and 1 μg/mL, to 1 μg/mL and 0.5 μg/mL for linezolid and vancomycin, respectively. These results showed that the bilberry leaves extract improves the antimicrobial activity of the tested antibiotics. The anti-staphylococcal activity might be linked to the phenolic compounds found in the leaves extracts that may increase the permeability of microbial cell walls or may deactivate the enzymes that act as protection for the microbial cells. 

The hydro-alcoholic extracts used by Cignarella et al. in their study have shown hypolipidemic activities in streptozotocin-induced diabetic rats [32]. The authors suggest that lipid lowering activity may be due the stimulation of the catabolism of the triglycerides-rich lipoproteins, and it can be performed in some circumstances, like insulin deficiency. According to these results, the bilberry extracts decreased the blood triglyceride levels of the dyslipidemic rats by 39%. Moreover, plasma glucose levels decreased by 26%.

The antioxidant capacity and antimicrobial activity of bilberry leaves was confirmed by several studies and these bioactive properties, attributed to proanthocyanidins, recommend applications for cosmetic, nutraceutical and pharmaceutical purposes [21,33,34].

### 2.2. Lingonberry Leaves

Lingonberry (*Vacccinium vitis-idaea* L.), also known as partridgeberry, foxberry or cowberry, is a small shrub native throughout North Eurasia’s and North America’s forests. Lingonberry plants are extremely hardy, tolerating −40 °C or lower temperatures with diverse habitats ranging from lowland to upland and mountain areas, and prefer acid soils [16]. The leaves of lingonberry remain green throughout winter, even in the coldest years, due to the snow covering them.

#### 2.2.1. Phenolic Content of the Lingonberry Leaves

Ek et al. detected 22 phenolic compounds in lingonberry samples (methanolic extracts from leaves and stems) [35]. Catechin, epicatechin and 5 catechin polymers (proanthocyanidins A and B) and flavonols, only conjugates of quercetin and kaempferol, have been identified in the samples. Furthermore, coumaroyl-hexose-hydroxyphenol, caffeoyl-hexose-hydroxyphenol and 2-*O*-caffeoyl-arbutin were identified as well; these components are distinct and were not found in bilberry leaves. Two flavonols, quercetin-3-*O*-(4″-HMG)-α-rhamnoside and kaempferol-HMG-rhamnoside were reported for the first time in this study [35].

Hokkanen et al. detected 36 phenolic compounds in the methanolic extract of lingonberry leaves using LC/TOF-MS and LC/MS/MS [25]. The main phenolic compounds detected were: quercetin-3-*O*-(4″-HMG)-α-rhamnoside, 2-*O*-caffeoylarbutin, quercetin-3-*O*-rutinoside (rutin), quercetin-3-*O*-β-galactoside, 10-*p*-trans-coumaroyl-1S-monotropein (coumaroyl iridoid 2), quercetin-3-*O*-α-rhamnoside (quercitrin), and kaempferol-HMG-rhamnoside with 32%, 10%, 8%, 6%, 6%, 5%, and 5% share, respectively, of the combined LC/TOF-MS peak areas of all detected phenolic compounds.

The methanolic extracts of lingonberry leaves revealed a higher flavonols content (66.9%), than phenolic acids content (24.4%). Regarding the flavonols, the one with the highest concentration was quercetin-3-*O*-(4″-HMG)-α-rhamnoside in both, methanolic and hydro-alcoholic extracts. From the group of phenolic acids chlorogenic acid, coumaroylquinic acid isomers, caffeic acid, *p*-coumaric acid, caffeoylshikimic acid and traces of one feruloylquinic acid isomer were found along with quercetin, quercetin glycosides, and some kaempferol glycosides. Lingonberry leaves also contain catechin, proanthocyanidins and coumaroyl iridoid 1 in low concentrations [25]. Arbutin derivatives like *p*-coumaroylarbutin, *p*-coumaroyl acetyl arbutin and caffeoyl acetyl arbutin were detected for the first time in hydro-alcoholic extracts of the lingonberry leaves by Ieri et al. [30]. On the other hand, Ieri et al. showed that hydroxycinnamic acids were found in higher proportions than flavonols in the hydro-alcoholic extracts.

Recently, Liu et al. identified 25 phenolic compounds in the lingonberry leaves extracts out of which quercetin-acetylglucoside and 2-*O*-caffeoyl-6-*O*-acetylarbutin were reported for the first time [27]. Other phenolic compounds reported in this study belong to the groups of phenolic acids, flavonols, flavanols and proanthocyanidins.

A complex study identified 90 phenolic compounds in lingonberry leaves [36]; the phenolic compounds included: (-)-epigallocathechin, some isomeric forms of cinchonains I and cinchonains II, two quercetin acetylhexosides, two sinapic acid hexosides and p-coumaroyl-dihydromonotropein, hydroxymethoxybenzoic acid hexoside, dihydroxybenzoic acid acetylhexoside, hydroxymethoxybenzoic acid acetylhexoside and hydroxymethoxyphenylacetic acid hexoside, were newly reported in lingonberry. Arbutin was the major compound detected in leaves, representing 31–50% of the total phenolic compounds, depending on the harvest period (May, July or September). The second and the third most abundant group of phenolic compounds were flavanols, with 27–42%, and flavonol glycosides, with 12–19% of the total phenolic content. Hydroxycinnamic acids were identified in lower quantities, ranging between 6–14% of the total of phenolic composition. Monomeric flavanols, like (+)-catechin and (-)-epicatechin and oligomeric flavanols with various structures, A-type dimers and trimers, and B-type dimers and trimers were identified along with some isomeric forms of cinchonains I and cinchonains II. Flavonol glycosides identified in the leaves of lingonberry were glycosides of quercetin and kaempferol, and quercetin aglycone as well. Caffeic acid derivatives, *p*-coumaric acid derivatives and sinapic acid derivatives represent the group of hydroxycinnamic acids present in lingonberry leaves.

Moreover, Vyas et al. conducted a comparative study between the leaves and fruits of lingonberry and showed that the content of total phenolic compounds, flavonoids, anthocyanins and tannins was considerably higher in the leaves compared to fruits [37]. Total radical scavenging capacity and reducing power were also higher in the leaves compared to the fruits. These results suggested that the powerful antioxidant activity of the leaves can be correlated with the high content of phenolic compounds.

#### 2.2.2. Therapeutic Uses and Biological Properties of the Lingonberry Leaves

Lingonberry leaves have similar properties to bilberry leaves, acting as diuretics and antiseptics on the urinary tract; these properties are associated with the high content of arbutin, arbutin derivatives and tannins [30,32]. Leaf extracts in different solvents have multiple properties; ethanolic extracts of lingonberry leaves have shown anti-cough activity, demonstrated by decreasing the frequency of coughing in ammonium-induced cough, phlegm-removing effect by increasing elimination of secretions from the trachea, and anti-inflammatory properties by decreasing the acetic acid-induced vascular permeability in rats [38].

Neuroprotective effect of the extracts was evidenced in vitro by Vyas et al. using acetone as the solvent [37]. Recent studies highlight that leaf extracts possess great reducing, radical scavenging and chelating activities and could be considered as a promising source of bioactive compounds with notable antioxidant activity [39].

### 2.3. Bog Bilberry Leaves

Bog bilberry (*Vaccinium uliginosum* L.) is a small shrub native to some regions of the Northern Hemisphere, especially at high altitudes, in zones of Europe, Asia and North America. It is an Arctic and boreal circumpolar species growing on wet acidic soils; both the leaves and fruits are consumed by many species of wildlife [16]. The phenolic composition of bog bilberry fruits has been extensively studied and the most important phenolic compounds treated were anthocyanins and flavonols [40,41,42]. In contrast, to the best of our knowledge, the phenolic composition of bog bilberry leaves is described only by one study [43].

#### Phenolic Content of the Bog Bilberry Leaves

Stanoeva et al. analyzed the phenolic composition in bog bilberry leaves, using methanolic extracts [43]. This study revealed 20 phenolic compounds in bog bilberry leaves from the groups of phenolic acids and derivatives, flavonols, flavan-3-ols, iridoids and cinchonain. The most important phenolic acid present in the leaf extract was identified as being chlorogenic acid (5-caffeoylquinic acid) representing 64% from the total phenolic acid content. Along with this compound other phenolic acids and derivatives were identified in the extract like 4-caffeoylquinic acid, *p*-coumaroylquinic acid derivatives (3-*p*-coumaroylquinic acid, 4-*p*-coumaroylquinic acid and 5-*p*-coumaroylquinic acid), feruloylquinic acid, gallic acid derivative and hexahydroxydiphenolyl-galloyl-glucose (HHDP-galloyl-glucose). This latter derivative was reported for the first time in *Vaccinium* species together with two flavonols, derivatives of kaempferol, kaempferol-feruloyl-acetylglucoside and kaempferol-coumaroylglucoside. The leaf extracts also revealed eight flavonols that were derivatives of quercetin, kaempferol and isorhamnetin. Along with these, other flavonols like kaempferol-3-*O*-arabinoside, quercetin-3-*O*-galactoside (hyperoside), quercetin-3-*O*-glucoside, quercetin-3-*O*-pentoside, quercetin-3-*O*-rhamnoside, and isorhamnetin-3-*O*-arabinoside were also detected in the leaves of bog bilberry. Procyanidin dimer was the only flavanol identified and two coumaroyl iridoid isomers, and one cinchonain compound was present in the leaves of bog bilberry.

### 2.4. Blueberry Leaves

Blueberry, *Vaccinium* spp. is a perennial shrub, popular around the world and comprise several related species, such as rabbit eye (*V*. *virgatum* L.), southern highbush (*V. formosum* L.) and lowbush (*V. angustifolium* L.). The most common blueberry worldwide is the northern highbush blueberry (*V. corymbosum* L.), also known as the blue huckleberry, tall huckleberry or high blueberry, which has higher levels of total anthocyanin content than other varieties [44]. Blueberry leaves are changing color from green to red in the autumn; consequently their phenolic composition is different during different seasons.

#### 2.4.1. Phenolic Content of the Blueberry Leaves

Riihinen et al. conducted a study to determine the phenolic components present in blueberry leaves [21]. The authors examined both green and red leaves and used methanol as extraction solvent. The results showed that the total phenolic content was similar in both green and red leaves, yet the individual composition exhibited interesting differences like the presence of anthocyanins in red leaves, only cyanidin-glycosides (62 μg/g frozen sample), and the absence in the green ones. The study revealed that the red leaves contain flavonols like quercetin and kaempferol, hydroxycinnamic acids like *p*-coumaric and caffeic or ferulic acids, and proanthocyanidins: prodelphinidins and procyanidins (Table 3). Green leaves had the same phenolic composition, but in lower concentrations, except for procyanidins where the concentration was higher [21].

Wang et al. studied 104 blueberry cultivars, including *V. corymbosum* [44]. All samples were analyzed with respect to their phenolic composition and antioxidant effect. Using HPLC-DAD and HPLC-ESI-MS on the methanolic extract, the authors identified anthocyanins, flavonols, hydroxycinnamic acids and proanthocyanidin in blueberry leaves, results that are in agreement with the study of Riihinen et al. [21]. The chlorogenic acids (esters of caffeic and quinic acid) were the most abundant phenolic compounds in the leaves of blueberry. The authors identified three anthocyanins in the blueberry leaves: cyanidin 3-*O*-glucoside, cyanidin 3-*O*-glucuronide and cyanidin 3-*O*-arabinoside, which provides the red color of the leaves.

Routray and Orsat conducted a study to check if the phytochemical composition and antioxidant activity of blueberry leaves are correlated with the harvest time [45]. The leaves of *V. corymbosum*, two varieties, Nelson and Elliot, were harvested at different periods of the year, respectively in May, July, September and October. The authors analyzed total phenolic content, total monomeric anthocyanin content and antioxidant activity. The total phenolic content was higher in the spring season (May), in July were lower than in May, after which the phenolic content started increasing in September and October. The highest quantity of phenolic compounds was observed in October (Table 4). The same pattern was observed for total monomeric anthocyanin content and for the antioxidant activity, for both varieties.

Based on the results the authors concluded that all properties were highest in blueberry leaves harvested in October, this being an optimal month for leaves harvest [45].

#### 2.4.2. Therapeutic Uses and Biological Properties of the Blueberry Leaves

The antimicrobial activity of the aqueous extracts of blueberry was shown against a panel of microorganisms. Silva et al. showed that the aqueous extract of blueberry leaves has antimicrobial activity against *S. aureus, Salmonella* Enteritidis, *Enterococcus faecium, Listeria innocua* and *Bacillus cereus* [46]. Antimicrobial activity was performed by well diffusion assay and was determined as MIC and MBC. Comparing the antimicrobial activity of blueberry leaves with that of the fruits it appeared that the leaf extracts had the highest activity. The MIC was 12.5 mg/mL or 25 mg/mL for the leaves, according to the bacterial strains and 50 mg/mL for the fruits, for all the tested bacterial strains [46].

Antimicrobial activity has also been investigated for hydroalcoholic blueberry leaves extracts [47]. The antimicrobial activity was determined by disk diffusion assay against some Gram-negative bacteria such as: *Escherichia coli, Pseudomonas aeruginosa, S. typhimurium, Acetobacter baumannii* and *Klebsiella pneumoniae* and Gram-positive bacteria, like: *S. aureus* and *Enterococcus faecalis.* The results exhibited that blueberry leaves extracts had antimicrobial activity against all tested bacteria. The diameters of the inhibition zone, expressed in mm, at a concentration of 5 mg/disc extract, were within the range 8.37, for *E. coli*, and 16.67, for *S. typhimurium.* Increasing the concentration to 10 mg/disc, the diameters of the inhibition zone increased, and were within the range 14.08, for *E. faecalis*, and 23.18 for *S. typhimurium* [47]. Given these results it cannot be clearly concluded whether the extracts have a greater effect on Gram positive or on Gram-negative microorganisms, however it is generally accepted that the main antimicrobial mechanism of phenolics is thought to be focused on the cytoplasmic membrane and therefore the outer lipidic membrane of the Gram negative bacteria may present an additional protection mechanism [43].

Furthermore, blueberry leaves decoction in distilled water (two hours at 100 °C) rich in hydroxycinnamic acids and quercetin glycosides had beneficial properties on the brain and liver of neonatal rats that were receiving toxic doses of selenium, to induce the oxidative stress [48]. Moreover blueberry leaves decoction prevented cataractogenesis in vivo and in vitro [49], these results pointing out the potential health promoting functions of blueberry leave extracts.

### 2.5. Bearberry Leaves

Bearberry (*Arctostaphylos uva-ursi* L.) is an evergreen shrub that is found in North America, Asia and Europe at high altitudes, also known as uva-ursi. The leaves are shiny, small, and feel thick and stiff, remaining green for 1–3 years before falling. The fruit is a red berry.

Bearberry leaves contain three major groups of phenolic compounds: phenols, tannins and flavonoids. The main and most important phenolic constituent present in bearberry leaves is arbutin (hydroquinone-β-ᴅ-glucopyranoside,5–15%), however, methylarbutin (up to 4%) and the free aglycones are considered also as main phenolic constituents. Other compounds found in the chemical profile are ursolic acid, tannic acid, gallic acid, *p*-coumaric acid, syringic acid, galloylarbutin, gallotannins (up to 20%) and from flavonoids, glycosides of quercetin, kaempferol and myricetin [50].

#### 2.5.1. Phenolic Content of the Bearberry Leaves

The leaves extracts of 14 Ericaceae species, including bearberry, were analyzed by RP-HPLC-DAD-APCI/MSD method by Saleem et al. [51]. The results of this study showed eight phenolic compounds present in the bearberry leaves extract: arbutin, catechin, myricitrin, quercetin-3-glucoside, procyanidin A2, one quercetin glycoside and two myricetin glycosides.

Furthermore, the results of Olennikov and Chekhirova [52] showed that bearberry leaves contain many phenolic compounds and the composition slightly differs from that shown by Saleem et al. [51]. According to Olennikov and Chekhirova, the dominant groups of phenolic compounds present in leaves were catechins (112.04 mg/g), simple phenols (97.38 mg/g) and tannins (72.58 mg/g). The three main phenolic compounds present in leaves were arbutin (82.4 mg/g), (+)-catechin (64.4 mg/g) and corilagin (57.51 mg/g). Other phenolic compounds found in leaves were: picein, some galloylglucose derivatives, chebulagic acid, caffeic acid and some of its derivatives, 5-caffeoylquinic acid (chlorogenic acid), cinnamic acid and two of its derivatives, isoferulic acid, some flavonoids, such as hyperoside, isoquercitrin, avicularin, quercitrin, rutin and quercetin-3-*O*-gentiobioside, and only traces of ferulic acid, (-)-epicatechin and quercetin. 

The chemical composition of bearberry leaves was also investigated by Panusa et al. [53]. The chemical composition determined by them was in agreement with the results obtained by Saleem et al. [51] and Olennikov and Chekhirova [52]. Some differences can be observed between the results of these studies for example 5-caffeoylquinic, caffeic, ferulic, isoferulic, cinnamic and methoxycinnamic acids were present in the leaves analyzed by Olennikov and Chekhirova [52], but were absent in the other two studies; another difference was that epicathechin, epigallocatechin and quercetin 3-*O*-gentiobioside detected in bearberry leaves by Olennikov and Chekhirova were not detected by Panusa et al. However, these differences can be attributed to a multitude of factors like season, region or exposure of the leaves, extraction or evaluation methods.

Parejo et al. pointed out, that arbutin levels in bearberry leaves collected during spring were slightly smaller (70.18 μg arbutin/mg dry weight) compared to leaves collected during autumn (82.18 μg arbutin/mg dry weight), therefore authors recommend leaves harvesting during autumn [54]. 

#### 2.5.2. Therapeutic Uses and Biological Properties of the Bearberry Leaves

Bearberry leave extracts are remedies for several affections. Amarowicz and Pegg showed the antiproliferative effects of bearberry leaves extracts against human carcinoma cell lines [55]. Phenolic compounds were isolated from the crude extract of bearberry leaves with 80% (v/v) aqueous ethanol and separated in two fractions, one fraction comprised low-molecular weight phenolics and the other one, high molecular weight phenolics (tannin fraction). The data revealed that the crude extract and its two fractions have the properties to inhibit the proliferation of five human carcinoma cell lines, namely MCF-7-breast, HT-29-colon, DU-145-prostate, SK-MEL-5-skin and MDA-MB-435-skin carcinoma. The antiproliferative activity was associated with gallotanins present in the bearberry leaves [55].

Bearberry leaves extracts have shown beneficial effects on diuresis and electrolyte composition of urine (excretion of K⁺ and Na⁺) on mice, after administration of 5% water extract of bearberry leaves [56]. The beneficial effects on diuresis of a bearberry extract were also exposed by Beaux et al. [57].

The antimicrobial activity of bearberry extract was tested by several studies [58,59]. According to Holopainen et al. the extracts of the aerial bearberry parts have antimicrobial activity against the Gram-negative bacteria: *E. coli* and *Proteus vulgaris*, effect attributed to arbutin and metylarbutin [58]. According to Annuk et al. [59], the aqueous extract of bearberry leaves had antimicrobial activity against *Helicobacter pylori* for which tannic acid was considered responsible. 

The most relevant health benefits of the five studied species are presented in Table 5.

## 3. The Major Bioactive Phenolic Compounds Present in Ericaceae Species Leaves 

Polyphenols in the diet are considered to be beneficial for human health and are attributed to anti-cancer and protective effects against cardiovascular diseases. Some of them can inhibit the activity of human topoisomerase II, overexpressed in highly proliferative tumor cells, and have been used as chemopreventive agent [60]. The major phenolic compounds identified in the leaves of the studied species are chlorogenic acid, quercetin and arbutin.

### 3.1. Chlorogenic Acid 

**Figure 1:**
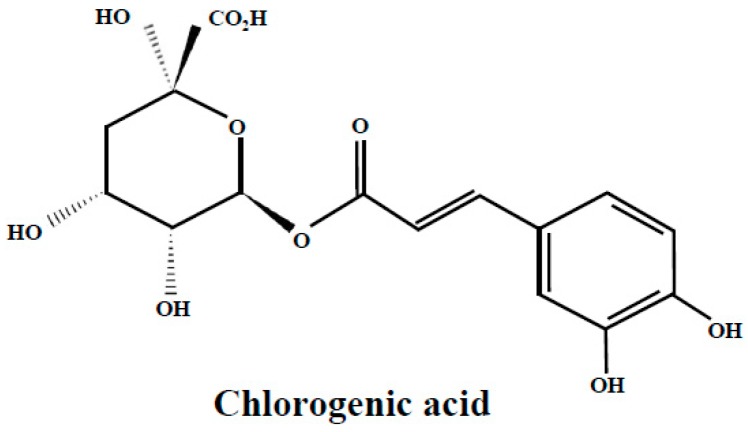
Chlorogenic acid structure.

Chlorogenic acid or 5-*O*-caffeoylquinic acid (Figure 1), the ester of caffeic acid with quinic acid, is one of the main phenolic compounds found in bilberry, bog bilberry and blueberry leaves [22,24,25,26,43,44]; it is also present in the leaves of lingonberry [25,30] and bearberry in smaller proportions [52]. Chlorogenic acid is a widespread phenolic compound in many plants and is one of the most abundant forms of phenolic compound in the human diet; it is extensively studied by many researchers and the results highlight multiple beneficial health effects via its antioxidative action. However, the mechanism of antioxidative action is not completely clarified. It is assumed that sequential proton loss electron transfer is the antioxidative mechanism of chlorogenic acid, in basic environment, and pathways like radical adduct formation and hydrogen atom transfer in acidic and neutral media [61].

Like other dietary polyphenols, chlorogenic acid exhibits various biological and pharmacological effects such as antioxidant activity [62,63], anti-obesity, anti-diabetic, anti-inflammatory and anti-lipidemic effects as well as antibacterial activity [64,65,66]. Shi et al. studied the effects of chlorogenic acid on hepatic damages and liver fibrosis induced by carbon tetrachloride in rats [67]. The histopathological examination showed general liver damages and fibrosis in the carbon tetrachloride administered group. On the other side, in the group treated with chlorogenic acid and carbon tetrachloride together, the liver fibrosis was developed in the periportal region, but it was inhibited in the pericentral region. These results clearly demonstrate the beneficial effect of chlorogenic acid against liver damages and fibrosis in rats. The hepatoprotective effect of chlorogenic acid was also confirmed in mice and the authors tested the effect of chlorogenic acid against acetaminophen induced liver injury [68]. All the negative effects of acetaminophen to the mice’s liver were prevented by administration of chlorogenic acid, especially at a concentration of 40 mg/kg.

Chlorogenic acid presented inhibitory activity against hepatitis B virus (HBV). The antiviral activity was tested in vitro, using HepG2.2.15 as model culture cells and in vivo using HBV- infected duck cells. The data collected from these studies demonstrated the antiviral activity of chlorogenic acid [69]. 

Chlorogenic acid has a significant renoprotective effect against kidney damages induced by administration of cisplatin on mice [70]. The mechanism of action of this effect was inhibition of renal oxidative stress, inflammation, apoptosis and autophagy.

Another beneficial effect of chlorogenic acid reported in literature is the anti-inflammatory activity. The results of the study conducted by Ruifeng et al. showed that chlorogenic acid is an effective anti-inflammatory agent and could reduce inflammation on lipopolysaccharide-induced mice mastitis [71].

The anti-inflammatory effect of chlorogenic acid was also reported by Zhang et al. [72]. The- authors showed the beneficial effect of chlorogenic acid against lipopolysaccharide-induced acute lung injury on mice.

### 3.2. Quercetin

**Figure 2:**
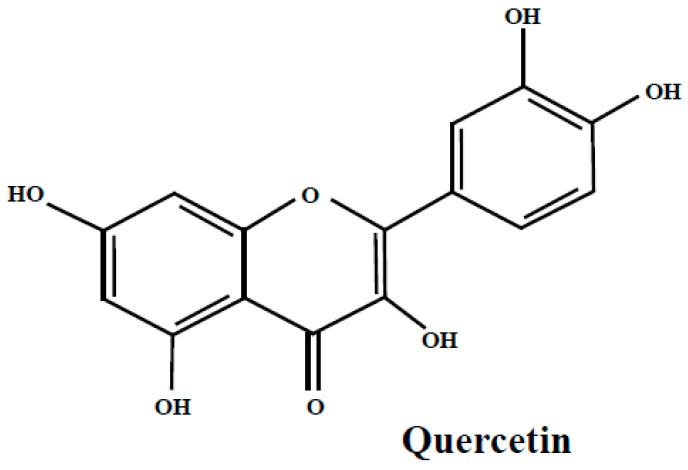
The quercetin structure.

Quercetin (Figure 2) is a flavonoid that occurs abundantly in many fruits and vegetables, with beneficial health impact. It is present in bilberry, bog bilberry, lingonberry, blueberry and bearberry leaves as quercetin or as quercetin glycosides [21,22,24,25,26,35,43,50,51,52]. Many studies were conducted on quercetin and its glycosides and most of them show their useful biological properties and therapeutic potency in relieving the symptoms of certain chronic diseases.

Duenas et al. tested the antioxidant activity for quercetin, catechin, epicatechin using FRAP and ABTS scavenging assay and α-tocopherol as control [73]. The results showed that the three tested flavonoids exercise better antioxidant activity than α-tocopherol, quercetin being the most potent compound.

The antioxidant activity of quercetin and its derivatives has been confirmed also in other studies [12,74]. Furthermore, Lesjak et al. suggested that quercetin has an anti-inflammatory effect, by inhibiting the synthesis of 12-HHT, TXB2 and PGE2 inflammatory mediators in a concentration-dependent manner [12].

Additionally, quercetin and quercetin-3-*O*-glycosides (guaijaverin, quercitrin, isoquercitrin, and hyperin) purified from *Bauhinia longifolia* leaves have shown anti-Mayaro virus (from the Togaviridae family, genus *Alphavirus*) activity. Quercetin alone exhibited an antiviral activity with 50% inhibitory concentration of viral replication (IC50) 10 ± 0.7 μg/mL and 90% inhibitory concentration of viral replication (IC90) 19 ± 0.7 μg/mL, greater than ribavirin (IC50 62 ± 4 and IC90 112 ± 8) used as positive control. However, ethyl acetate and n-butanol fractions containing a mixture of quercetin and its four glycosides exposed a more potent antiviral activity than those seen on quercetin and ribavirin, respectively, IC50 5 ± 0.3 μg/mL; IC90 25 ± 3.5 μg/mL for ethyl acetate fraction and IC50 3 ± 0.2 μg/mL; IC90 5 ± 0.3 μg/mL for n-butanol fraction [75].

Quercetin has multiple pathways that lead to the removal of cancer cells, respectively, inhibit cell growth and angiogenesis, induce apoptosis, either by downregulation of oncogenes, or through upregulation of tumor suppressor genes and cellular senescence. It has been reported to inhibit cell proliferation in MDA-MB-231 human breast cancer [76], MCF-7 breast cancer [77], SW480 colon cancer [78] and HT-2 colon cancer [79]. Similarly, quercetin had a beneficial effect in A549 lung cancer cells and in a human prostate cell line (LNCaP), inducing apoptosis of cancer cells [80,81]. Recently, the effects of quercetin on cancer cells were also analyzed in other types of cancer: pancreatic cancer in vivo and in vitro [82], ovarian cancer [83] and gastric cancer [84]. In addition to all these important and beneficial effects quercetin has also cardioprotective activity which may be attributed to its anti-inflammatory and antioxidant properties [85,86].

### 3.3. Arbutin

**Figure 3:**
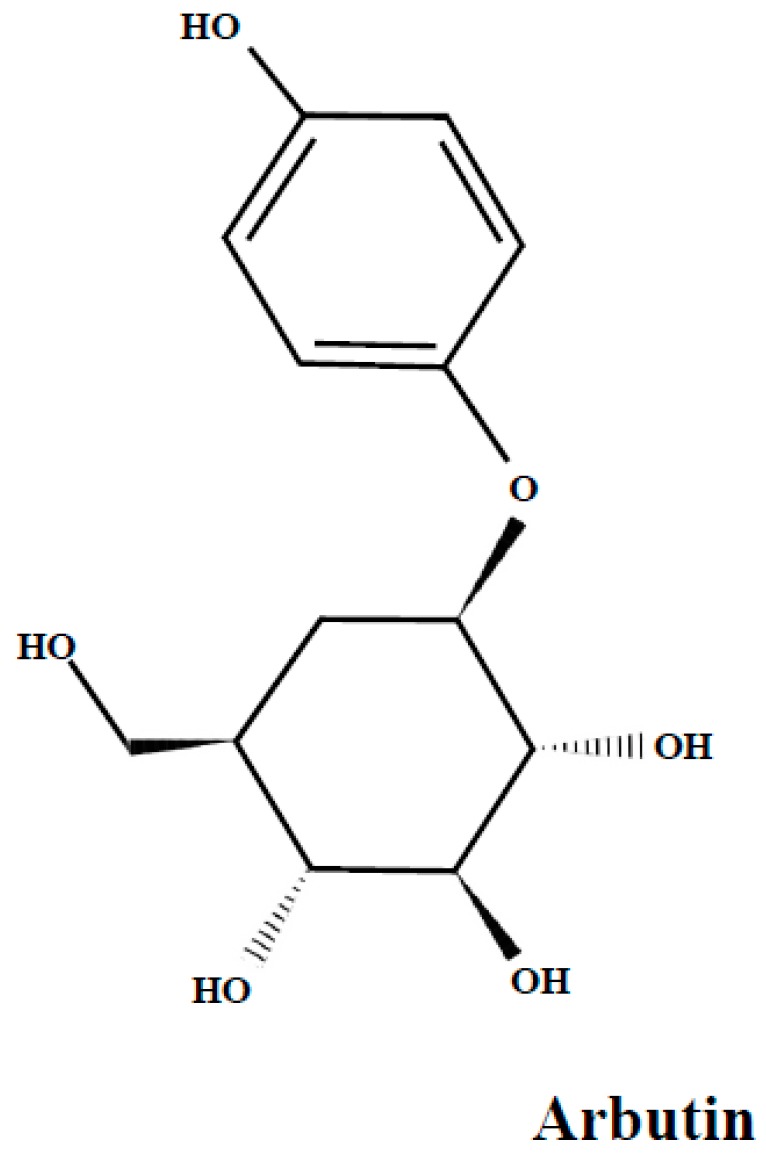
The arbutin structure.

Arbutin (Figure 3), is a glucoside of hydroquinone. Its derivatives are phenolic compounds present in the bearberry leaves as dominant compounds [50,51] and they are also found in lingonberry leaves [25,30]. 

One of the most important properties of arbutin is its widespread use as a skin-whitening agent, an action that occurs due to inhibition of the enzyme tyrosinase, the enzyme implicated in melanogenesis [87].

Furthermore, another biological activity of arbutin consists in its efficacy in the treatment of various urinary tract infections due to the antibacterial, astringent, antioxidant and disinfectant effect of hydroquinone, the active metabolite of arbutin [88,89,90]. This compound has a disinfectant effect on the urinary tract due to hydrolysis of hydroquinone, but only in an alkaline environment. In addition, arbutin validated its antioxidant activity through an ABTS radical cation-scavenging assay; the results showed similar or higher antioxidant activity than hydroquinone, an efficient antioxidant. In fact, one molecule of arbutin scavenged three molecules of ABTS radical cation, while hydroquinone scavenged two molecules of the same radical cation [91]. The antioxidant activity of arbutin was also determined in other studies by different methods of analysis [92,93,94]. Moreover, some in vitro studies have been conducted and showed the cytotoxic effect [95,96] and the anti-inflammatory properties of arbutin [97,98].

## 4. Bioaccessibility and Bioavailability of Key Compounds

Bioaccessibility can be defined as the amount or fraction of a compound which is released into the gastrointestinal tract and becomes available for absorption [99]. To determine the bioaccessibility of a compound, different in vitro digestion methods can be used (the most commonly used are the small intestine and gastric digestion simulations). In vitro digestion methods consist of three stages: release of the compound from the matrix, its modification due to digestion conditions and the absorption of the intestinal epithelium cells [100].

Bioavailability is defined as the amount of a compound that is utilized after its assimilation in systemic circulation [101]. The bioavailability of a compound can be established using in vivo tests on animals or humans. In vivo determinations of bioavailability include digestion in the gastrointestinal tract, assimilation, metabolism, circulation of the compound or its metabolites to the tissues and excretion [100].

Polyphenols represent a large and various group of compounds and their bioavailability may vary within the group. The physicochemical properties of phenolic compounds, such as: structure and molecular size, solubility, degree of polymerization, conjugation or glycosylation, influence their absorption and metabolism. Therefore, phenolic compounds with low molecular weight are quickly absorbed, while large polyphenols with high molecular weight are poorly absorbed [100]. The free form of phenolic compounds (the aglycones) can be easily absorbed from the small intestine. In fact, many polyphenols are found in conjugate forms, such as esters, glycosides or polymers and they must be metabolized in order to be absorbed. The metabolism of conjugate forms is accomplished through hydrolysis by intestinal enzymes or by the colonic microbiota. When phenolic compounds are hydrolyzed by the colonic microbiota, their absorption can be reduced because the microorganisms can also degrade the aglycones resulted from the hydrolysis of the phenolic compounds, leading to simple aromatic acids. During absorption, polyphenols suffer certain modifications in the intestine or in the liver, such as methylation, sulfation and glucuronidation. Afterwards, polyphenols and their metabolites are released, especially in the bile or urine. From the bile, polyphenols are secreted into duodenum, where bacterial enzymes act on them, and finally can be reabsorbed [6].

Phenolic acids are a specific class of polyphenols which are usually involved in mechanisms of defense against biotic and abiotic stress [102]. Gallic acid is a phenolic acid, representing the hydroxybenzoic group and in terms of bioavailability it is a very well absorbed acid. Scientific literature showed that gallic acid can be absorbed in the stomach, small intestine or both. After ingestion, either in a food matrix or in free form, gallic acid is transformed and can be found in plasma and urine, mainly in the 4-*O*-methylated form and *O*-glucuronidated form. Other hydroxybenzoic acids include ellagic acid, protocatechuic acid and 4-hydrobenzoic acid. Phenolic acids from the hydroxycinnamic group occur in higher concentrations in foods than the hydroxybenzoic group. Caffeic acid, *p*-coumaric acid, sinapic acid and ferulic acid are representatives for the group of hydroxycinnamic acids. The free form of hydroxycinnamic acids is quickly absorbed from the stomach and small intestine. Afterwards, hydroxycinnamates are conjugated by enzymes (intestinal and hepatic detoxification enzymes) [103]. The esterified forms, with quinic acid, tartaric acid or carbohydrate derivatives are digested in the colon [104].

Flavonoids represent an extensive group of polyphenols, containing more than six thousand compounds. Flavonoids are mainly bonded to β-glycoside sugars in plants. The metabolization of flavonoids begins with hydrolysis of the glycosidic linkage, action that takes place in the intestine lumen or in enterocytes, immediately preceding the absorption. Flavonoids linked to glucose, arabinose or xylose are hydrolyzed by human endogenous cytosolic β-glucosidase. Instead, flavonoids linked with rhamnose are hydrolyzed only by rhamnosidase, an enzyme produced by intestinal microbiota [105].

## 5. Conclusions

The Ericaceae family contains various plant species with traditional therapeutic use against several diseases. Phytochemicals like flavonoids, phenolic acids, and vitamins are thought to be the trigger bioactives that prevent inflammation, oxidative stress and the installation of several non-communicable diseases. 

Leaves of berries contain plentiful bioactive compounds, which are mostly polyphenols. However, the number of studies on leaf extracts are considerably fewer than the studies conducted on berry fruit extracts. The three predominant phytochemicals found in the leaves of the studied species are quercetin, chlorogenic acid and arbutin, these compounds validated their health benefits in numerous in vitro and in vivo studies. The chemical composition of wild and cultivated berries shows a more complex profile of the wild berries compared to the cultivated species. Regarding the bioavailability studies, it can be concluded that large polyphenols with high molecular weights are poorly absorbed in the organism and the conjugate forms, such as esters, glycosides or polymers must be metabolized first in order to be absorbed. 

Evaluation of the health effects of natural bioactive components is an intensively studied topic, but, their mechanisms of action and the inter-relations between them like synergism, antagonisms and others have not been clearly evidenced and further research is needed to elucidate this inconsistency. 

## Figures and Tables

**Table 1:** The content of flavonols, hydroxycinnamic acids and proanthocyanidins in bilberry leaves.

Phenolic Compounds		Content in the Red Leaves (μg/g)	Content in the Green Leaves (μg/g)
Flavonols	Quercetin	10,369	3369
	kaempferol	244	171
Hidroxycinnamic Acids	p-coumaric	6007	2989
	caffeic or ferulic	16,249	7808
Proanthocyanidins	prodelphinidins	36	25
	procyanidins	402	962

**Table 2:** The content of caffeic acid and coumaric acid derivatives in bilberry leaves according to the harvest period.

Harvest Period	Year of Harvest	Caffeic Acid Derivatives (mg/g Dry Extract)	Coumaric Acid Derivatives (mg/g Dry Extract)
May	2013	65.2 ± 5.6	21.6 ± 2.3
	2014	124.6 ± 3.5	35.8 ± 1.4
July	2013	98.0 ± 10.6	8.83 ± 0.78
	2014	100.5 ± 0.6	10.2 ± 0.0
September	2013	72.1 ± 4.4	7.48 ± 0.25
	2014	72.3 ± 0.7	7.91 ± 0.21

**Table 3:** The content of flavonols, hydroxycinnamic acids and proanthocyanidins in blueberry leaves.

Phenolic Compounds		Content in the Red Leaves (μg/g)	Content in the Green Leaves (μg/g)
**Flavonols**	Quercetin	3530	1784
	Kaempferol	505	191
Hidroxycinnamic acids	p-coumaric	3060	490
	caffeic or ferulic acid	19,870	7537
Proanthocyanidins	Prodelphinidins	485	468
	Procyanidins	272	364

**Table 4:** Total phenolic content, total monomeric anthocyanin content and antioxidant activity of the blueberry leaves (Nelson and Elliot varieties) according to the harvest period.

Harvest month	Total Phenolic Content (mg GAE */g Dry Matter)		Total Monomeric Anthocyanin (mg M3GE */g Dry Matter)		% Inhibition DPPH *	
	Nelson Variety	Elliot Variety	Nelson Variety	Elliot Variety	Nelson Variety	Elliot Variety
May	106.901 ± 2.765	123.749 ± 2.473	0.548 ± 0.018	0.221 ± 0.042	81.55 ± 0.94	85.91 ± 0.86
July	86.457 ± 0.741	106.133 ± 0.962	0.366 ± 0.179	0.245 ± 0.075	73.54 ± 1.94	84.32 ± 0.30
September	105.204 ± 3.826	120.962 ± 1.420	0.742 ± 0.015	0.135 ± 0.106	82.94 ± 0.11	85.43 ± 0.37
October	152.356 ± 3.369	155.830 ± 2.103	1.202 ± 0.080	0.714 ± 0.115	89.17 ± 0.24	88.09 ± 0.23

* GAE-gallic acid equivalent; M3GE-malvidin 3-glucoside equivalent, DPPH-2,2-diphenyl-1-picrylhydrazyl.

**Table 5:** Several properties and health benefits of the five studied species.

Studied Species	Properties	Health Benefits	References
Bilberry leaves	Astringent, antiseptic	Traditionally used to treat the affections of urinary tract	[29]
		Anti-bacterial effect	[31,34]
		Anti-inflammatory	[29]
	Lipid-lowering	Decreased blood triglycerides level	[32]
	Antioxidant		[34]
Lingonberry leaves	Diuretic, Antiseptic	Treatment of the urinary tract	[30,32]
	Phlegm-removing effect	Anti-cough activity	[38]
		Anti-inflammatory properties	[38]
		Neuroprotective effect	[37]
	Antioxidant		[36,39]
Bog bilberry leaves	Antioxidant		[41,42]
Blueberry leaves		Anti-microbial activity	[46,47]
	Antioxidant	Hepato-protective and neuroprotective effect	[45,47,48,49]
		Prevention of cataractogenesis	[49]
Bear berry leaves	Antiproliferative	Inhibition of human carcinoma cell lines	[55]
		Beneficial effect on diuresis and electrolyte composition	[56,57]
		Anti-microbial activity	[58,59]

## Supplementary Materials

The following are available online at https://www.mdpi.com/1420-3049/24/11/2046/s1.

## References

[1] Christenhusz M.J.M. (2016). The Number of known Plants Spesies in the Word and its Annual Increase. Phytotaxa.

[2] Martins N., Barros L., Ferreira I.C.F.R. (2016). In vivo antioxidant activity of phenolic compounds: Facts and gaps. Trends Food Sci. Technol..

[3] Spencer J.P.E., Crozier A., Rodriguez-Mateos A., Del Rio D., Tognolini M., Borges G. (2012). Dietary (Poly)phenolics in Human Health: Structures, Bioavailability, and Evidence of Protective Effects Against Chronic Diseases. Antioxid. Redox Signal..

[4] Shahidi F., Ambigaipalan P. (2015). Phenolics and polyphenolics in foods, beverages and spices: Antioxidant activity and health effects—A review. J. Funct. Foods.

[5] Bravo L. (1998). Polyphenols: Chemistry, Dietary Sources, Metabolism, and Nutritional Significance. Nutr. Rev..

[6] Manach C., Scalbert A., Morand C., Rémésy C., Jiménez L. (2004). Polyphenols: Food sources and bioavailability. Am. J. Clin. Nutr..

[7] Manach C., Williamson G., Morand C., Scalbert A. (2005). Bioavailability and bioefficacy of polyphenols in humans. I. Review of 97 bioavailability studies. Am. J. Clin. Nutr..

[8] Prior R.L., Cao G., Martin A., Sofic E., McEwen J., O’Brien C., Lischner N., Ehlenfeldt M., Kalt W., Krewer G. (1998). Antioxidant Capacity As Influenced by Total Phenolic and Anthocyanin Content, Maturity, and Variety of Vaccinium Species. J. Agric. Food Chem..

[9] Moyer R.A., Hummer K.E., Finn C.E., Frei B., Wrolstad R.E. (2002). Anthocyanins, Phenolics, and Antioxidant Capacity in Diverse Small Fruits: *Vaccinium*, *Rubus*, and *Ribes*. J. Agric. Food Chem..

[10] Taruscio T.G., Barney D.L., Exon J. (2004). Content and Profile of Flavanoid and Phenolic Acid Compounds in Conjunction with the Antioxidant Capacity for a Variety of Northwest Vaccinium Berries. J. Agric. Food Chem..

[11] Zafra-stone S., Yasmin T., Bagchi M., Chatterjee A. (2007). Berry anthocyanins as novel antioxidants in human health and disease prevention. Mol. Nutr. Food Res..

[12] Lesjak M., Beara I., Simin N., Pintać D., Majkić T., Bekvalac K., Orčić D., Mimica-Dukić N. (2018). Antioxidant and anti-inflammatory activities of quercetin and its derivatives. J. Funct. Foods.

[13] Márquez-García B., Fernández M.Á., Córdoba F. (2009). Phenolics composition in Erica sp. differentially exposed to metal pollution in the Iberian Southwestern Pyritic Belt. Bioresour. Technol..

[14] Diaconeasa Z. (2018). Time-Dependent Degradation of Polyphenols from Thermally-Processed Berries and Their In Vitro Antiproliferative Effects against Melanoma. Molecules.

[15] Bunea A., Ruginǎ D.O., Pintea A.M., Sconţa Z., Bunea C.I., Socaciu C. (2011). Comparative polyphenolic content and antioxidant activities of some wild and cultivated blueberries from romania. Not. Bot. Horti Agrobot. Cluj-Napoca.

[16] Chittaranjan K. (2011). Wild Crop Relatives: Genomic and Breeding Resources, Temperate Fruits.

[17] Nardini M., Ghiselli A. (2004). Determination of free and bound phenolic acids in beer. Food Chem..

[18] D’Archivio M., Filesi C., Di Benedetto R., Gargiulo R., Giovannini C., Masella R. (2007). Polyphenols, dietary sources and bioavailability. Ann. Ist. Super. Sanita.

[19] Häkkinen S.H., Kärenlampi S.O., Heinonen I.M., Mykkänen H.M., Törronen A.R. (1999). Content of the flavonols quercetin, myricetin, and kaempferol in 25 edible berries. J. Agric. Food Chem..

[20] Määttä-Riihinen K.R., Kamal-Eldin A., Mattila P.H., González-Paramás A.M., Törrönen R. (2004). Distribution and contents of phenolic compounds in eighteen scandinavian berry species. J. Agric. Food Chem..

[21] Riihinen K., Jaakola L., Kärenlampi S., Hohtola A. (2008). Organ-specific distribution of phenolic compounds in bilberry (Vaccinium myrtillus) and “northblue” blueberry (Vaccinium corymbosum x V. angustifolium). Food Chem..

[22] Fraisse D., Carnat A., Lamaison J.L. (1996). Composition polyphenolique de la feuille de myrtille. Ann. Pharm. Fr..

[23] Jaakola L., Määttä-Riihinen K., Kärenlampi S., Hohtola A. (2004). Activation of flavonoid biosynthesis by solar radiation in bilberry (Vaccinium myrtillus L.) leaves. Planta.

[24] Oszmiański J., Wojdyło A., Gorzelany J., Kapusta I. (2011). Identification and characterization of low molecular weight polyphenols in berry leaf extracts by HPLC-DAD and LC-ESI/MS. J. Agric. Food Chem..

[25] Hokkanen J., Mattila S., Jaakola L., Pirttilä A.M., Tolonen A. (2009). Identification of phenolic compounds from lingonberry (Vaccinium vitis-idaea L.), Bilberry (Vaccinium myrtillus L.) andHybrid Bilberry (Vaccinium x intermedium Ruthe L.) Leaves. J. Agric. Food Chem..

[26] Martz F., Jaakola L., Julkunen-Tiitto R., Stark S. (2010). Phenolic Composition and Antioxidant Capacity of Bilberry (Vaccinium myrtillus) Leaves in Northern Europe Following Foliar Development and Along Environmental Gradients. J. Chem. Ecol..

[27] Liu P., Lindstedt A., Markkinen N., Sinkkonen J., Suomela J.-P., Yang B. (2014). Characterization of Metabolite Profiles of Leaves of Bilberry (*Vaccinium myrtillus* L.) and Lingonberry (*Vaccinium vitis-idaea* L.). J. Agric. Food Chem..

[28] Bujor O.C., Le Bourvellec C., Volf I., Popa V.I., Dufour C. (2016). Seasonal variations of the phenolic constituents in bilberry (Vaccinium myrtillus L.) leaves, stems and fruits, and their antioxidant activity. Food Chem..

[29] Fernando P. (2007). Compendio di gemmoterapia clinica.

[30] Ieri F., Martini S., Innocenti M., Mulinacci N. (2013). Phenolic distribution in liquid preparations of Vaccinium myrtillus L. and Vaccinium vitis idaea L.. Phytochem. Anal..

[31] Sadowska B., Paszkiewicz M., Podsȩdek A., Redzynia M., Rózalska B. (2014). Vaccinium myrtillus leaves and Frangula alnus bark derived extracts as potential antistaphylococcal agents. Acta Biochim. Pol..

[32] Cignarella A., Nastasi M., Cavalli E., Puglisi L. (1996). Novel lipid-lowering properties of Vaccinium myrtillus L. leaves, a traditional antidiabetic treatment, in several models of rat dyslipidaemia: A comparison with ciprofibrate. Thromb. Res..

[33] Piljac-Žegarac J., Belščak A., Piljac A. (2009). Antioxidant capacity and polyphenolic content of blueberry (Vaccinium corymbosum L.) leaf infusions. J. Med. Food.

[34] Heinonen M. (2007). Antioxidant activity and antimicrobial effect of berry phenolics—A Finnish perspective. Mol. Nutr. Food Res..

[35] Ek S., Kartimo H., Mattila S., Tolonen A. (2006). Characterization of Phenolic Compounds from Lingonberry (*Vaccinium vitis-idaea*). J. Agric. Food Chem..

[36] Bujor O.C., Ginies C., Popa V.I., Dufour C. (2018). Phenolic compounds and antioxidant activity of lingonberry (Vaccinium vitis-idaea L.) leaf, stem and fruit at different harvest periods. Food Chem..

[37] Vyas P., Kalidindi S., Chibrikova L., Igamberdiev A.U., Weber J.T. (2013). Chemical analysis and effect of blueberry and lingonberry fruits and leaves against glutamate-mediated excitotoxicity. J. Agric. Food Chem..

[38] Wang X., Sun H., Fan Y., Li L., Makino T., Kano Y. (2005). Analysis and bioactive evaluation of the compounds absorbed into blood after oral administration of the extracts of Vaccinium vitis-idaea in rat. Biol. Pharm. Bull..

[39] Raudone L., Vilkickyte G., Pitkauskaite L., Raudonis R., Vainoriene R., Motiekaityte V. (2019). Antioxidant Activities of Vaccinium vitis-idaea L. Leaves within Cultivars and Their Phenolic Compounds. Molecules.

[40] Lätti A.K., Jaakola L., Riihinen K.R., Kainulainen P.S. (2010). Anthocyanin and flavonol variation in bog bilberries (Vaccinium uliginosum L.) in Finland. J. Agric. Food Chem..

[41] Wang L.J., Su S., Wu J., Du H., Li S.S., Huo J.W., Zhang Y., Wang L.S. (2014). Variation of anthocyanins and flavonols in Vaccinium uliginosum berry in Lesser Khingan Mountains and its antioxidant activity. Food Chem..

[42] Su S., Wang L.J., Feng C.Y., Liu Y., Li C.H., Du H., Tang Z.Q., Xu Y.J., Wang L.S. (2016). Fingerprints of anthocyanins and flavonols of Vaccinium uliginosum berries from different geographical origins in the Greater Khingan Mountains and their antioxidant capacities. Food Control.

[43] Stanoeva J.P., Stefova M., Andonovska K.B., Vankova A., Stafilov T. (2017). Phenolics and mineral content in bilberry and bog bilberry from Macedonia. Int. J. Food Prop..

[44] Wang L.-J., Wu J., Wang H.-X., Li S.-S., Zheng X.-C., Du H., Xu Y.-J., Wang L.-S. (2015). Composition of phenolic compounds and antioxidant activity in the leaves of blueberry cultivars. J. Funct. Foods.

[45] Routray W., Orsat V. (2014). Variation of phenolic profile and antioxidant activity of North American highbush blueberry leaves with variation of time of harvest and cultivar. Ind. Crops Prod..

[46] Silva S., Costa E.M., Pereira M.F., Costa M.R., Pintado M.E. (2013). Evaluation of the antimicrobial activity of aqueous extracts from dry Vaccinium corymbosum extracts upon food microorganism. Food Control.

[47] Pervin M., Hasnat M.A., Lim B.O. (2013). Antibacterial and antioxidant activities of Vaccinium corymbosum L. leaf extract. Asian Pacific J. Trop. Dis..

[48] Ferlemi A.V., Mermigki P.G., Makri O.E., Anagnostopoulos D., Koulakiotis N.S., Margarity M., Tsarbopoulos A., Georgakopoulos C.D., Lamari F.N. (2015). Cerebral Area Differential Redox Response of Neonatal Rats to Selenite-Induced Oxidative Stress and to Concurrent Administration of Highbush Blueberry Leaf Polyphenols. Neurochem. Res..

[49] Ferlemi A.V., Makri O.E., Mermigki P.G., Lamari F.N., Georgakopoulos C.D. (2016). Quercetin glycosides and chlorogenic acid in highbush blueberry leaf decoction prevent cataractogenesis in vivo and in vitro: Investigation of the effect on calpains, antioxidant and metal chelating properties. Exp. Eye Res..

[50] Barl B., Loewen D., Svendsen E. (1996). Arctostaphylos uva ursi L. Spreng. Saskatchewan Herb Database.

[51] Saleem A., Harris C.S., Asim M., Cuerrier A., Martineau L., Haddad P.S., Arnason J.T. (2010). A RP-HPLC-DAD-APCI/MSD method for the characterisation of medicinal Ericaceae used by the Eeyou Istchee Cree First Nations. Phytochem. Anal..

[52] Olennikov D.N., Chekhirova G.V. (2013). 6″-Galloylpicein and other phenolic compounds from Arctostaphylos uva-ursi. Chem. Nat. Compd..

[53] Panusa A., Petrucci R., Marrosu G., Multari G., Gallo F.R. (2015). UHPLC-PDA-ESI-TOF/MS metabolic profiling of Arctostaphylos pungens and Arctostaphylos uva-ursi. A comparative study of phenolic compounds from leaf methanolic extracts. Phytochemistry.

[54] Parejo I., Viladomat F., Bastida J., Codina C. (2001). A single extraction step in the quantitative analysis of arbutin in bearberry (Arctostaphylos uva-ursi) leaves by high-performance liquid chromatography. Phytochem. Anal..

[55] Amarowicz R., Pegg R.B. (2013). Inhibition of proliferation of human carcinoma cell lines by phenolic compounds from a bearberry-leaf crude extract and its fractions. J. Funct. Foods.

[56] Vranješ M., Popović B.M., Štajner D., Ivetić V., Mandić A., Vranješ D. (2016). Effects of bearberry, parsley and corn silk extracts on diuresis, electrolytes composition, antioxidant capacity and histopathological features in mice kidneys. J. Funct. Foods.

[57] Beaux D., Fleurentin J., Mortier F. (1999). Effect of extracts of Orthosiphon stamineus Benth, Hieracium pilosella L., Sambucus nigra L. and Arctostaphylos uva-ursi (L.) Spreng. in rats. Phyther. Res..

[58] Holopainen M., Jahodar L., Seppänen-Laakso T., Laakso I., Kauppinen V. (1988). Antimicrobial activity of some Finnish Ericaceous plants. Acta Pharm. Fenn..

[59] Annuk H., Hirmo S., Türi E., Mikelsaar M., Arak E., Wadström T. (1999). Effect on cell surface hydrophobicity and susceptibility of Helicobacter pylori to medicinal plant extracts. FEMS Microbiol. Lett..

[60] Mejı D., Gonza E., Chandra S., Ramı M., Wang W. (2006). Catalytic inhibition of human DNA topoisomerase by phenolic compounds in Ardisia compressa extracts and their effect on human colon cancer cells. Food Chem. Toxicol..

[61] Markovic S., Dimitric J.M., Mojovic M., Milenkovic D., Tošovic J. (2017). Antioxidative mechanisms in chlorogenic acid. Food Chem..

[62] Marinova E.M., Toneva A., Yanishlieva N. (2009). Comparison of the antioxidative properties of caffeic and chlorogenic acids. Food Chem..

[63] Sato Y., Itagaki S., Kurokawa T., Ogura J., Kobayashi M., Hirano T., Sugawara M., Iseki K. (2011). In vitro and in vivo antioxidant properties of chlorogenic acid and caffeic acid. Int. J. Pharm..

[64] Cho A.S., Jeon S.M., Kim M.J., Yeo J., Seo K.I., Choi M.S., Lee M.K. (2010). Chlorogenic acid exhibits anti-obesity property and improves lipid metabolism in high-fat diet-induced-obese mice. Food Chem. Toxicol..

[65] Ong K.W., Hsu A., Tan B.K.H. (2013). Anti-diabetic and anti-lipidemic effects of chlorogenic acid are mediated by ampk activation. Biochem. Pharmacol..

[66] Lou Z., Wang H., Zhu S., Ma C., Wang Z. (2011). Antibacterial activity and mechanism of action of chlorogenic acid. J. Food Sci..

[67] Shi H., Dong L., Bai Y., Zhao J., Zhang Y., Zhang L. (2009). Chlorogenic acid against carbon tetrachloride-induced liver fibrosis in rats. Eur. J. Pharmacol..

[68] Ji L., Jiang P., Lu B., Sheng Y., Wang X., Wang Z. (2013). Chlorogenic acid, a dietary polyphenol, protects acetaminophen-induced liver injury and its mechanism. J. Nutr. Biochem..

[69] Wang G.F., Shi L.P., Ren Y.D., Liu Q.F., Liu H.F., Zhang R.J., Li Z., Zhu F.H., He P.L., Tang W. (2009). Anti-hepatitis B virus activity of chlorogenic acid, quinic acid and caffeic acid in vivo and in vitro. Antiviral Res..

[70] Domitrović R., Cvijanović O., Šušnić V., Katalinić N. (2014). Renoprotective mechanisms of chlorogenic acid in cisplatin-induced kidney injury. Toxicology.

[71] Ruifeng G., Yunhe F., Zhengkai W., Ershun Z., Yimeng L., Minjun Y., Xiaojing S., Zhengtao Y., Naisheng Z. (2014). Chlorogenic acid attenuates lipopolysaccharide-induced mice mastitis by suppressing TLR4-mediated NF-κB signaling pathway. Eur. J. Pharmacol..

[72] Zhang X., Huang H., Yang T., Ye Y., Shan J., Yin Z., Luo L. (2010). Chlorogenic acid protects mice against lipopolysaccharide-induced acute lung injury. Injury.

[73] Dueñas M., González-Manzano S., González-Paramás A., Santos-Buelga C. (2010). Antioxidant evaluation of O-methylated metabolites of catechin, epicatechin and quercetin. J. Pharm. Biomed. Anal..

[74] Wiczkowski W., Szawara-Nowak D., Topolska J., Olejarz K., Zieliński H., Piskuła M.K. (2014). Metabolites of dietary quercetin: Profile, isolation, identification, and antioxidant capacity. J. Funct. Foods.

[75] Dos Santos A.E., Kuster R.M., Yamamoto K.A., Salles T.S., Campos R., De Meneses M.D.F., Soares M.R., Ferreira D. (2014). Quercetin and quercetin 3-O-glycosides from Bauhinia longifolia (Bong.) Steud. show anti-Mayaro virus activity. Parasites Vectors.

[76] Conklin C.M.J., Bechberger J.F., MacFabe D., Guthrie N., Kurowska E.M., Naus C.C. (2007). Genistein and quercetin increase connexin43 and suppress growth of breast cancer cells. Carcinogenesis.

[77] Deng X.H., Song H.Y., Zhou Y.F., Yuan G.Y., Zheng F.J. (2013). Effects of quercetin on the proliferation of breast cancer cells and expression of survivin in vitro. Exp. Ther. Med..

[78] Shan B.E., Wang M.X., Li R.Q. (2009). Quercetin inhibit human SW480 colon cancer growth in association with inhibition of cyclin D1 and survivin expression through Wnt/β-catenin signaling pathway. Cancer Invest..

[79] Lee Y.-K., Park S.Y., Kim Y.-M., Lee W.S., Park O.J. (2009). AMP kinase/cyclooxygenase-2 pathway regulates proliferation and apoptosis of cancer cells treated with quercetin. Exp. Mol. Med..

[80] Nguyen T.T.T., Tran E., Nguyen T.H., Do P.T., Huynh T.H., Huynh H. (2004). The role of activated MEK-ERK pathway in quercetin-induced growth inhibition and apoptosis in A549 lung cancer cells. Carcinogenesis.

[81] Kim Y.H., Lee Y.J. (2007). TRAIL apoptosis is enhanced by quercetin through Akt dephosphorylation. J. Cell. Biochem..

[82] Angst E., Park J.L., Moro A., Lu Q.-Y., Lu X., Li G., King J., Chen M., Reber H.A., Go V.L.W. (2013). The flavonoid quercetin inhibits pancreatic cancer growth in vitro and in vivo. Pancreas.

[83] Maciejczyk A., Surowiak P. (2013). Quercetin inhibits proliferation and increases sensitivity of ovarian cancer cells to cisplatin and paclitaxel. Ginekol. Pol..

[84] Ramachandran L., Manu K.A., Shanmugam M.K., Li F., Siveen K.S., Vali S., Kapoor S., Abbasi T., Surana R., Smoot D.T. (2012). Isorhamnetin inhibits proliferation and invasion and induces apoptosis through the modulation of peroxisome proliferator-activated receptor γ activation pathway in gastric cancer. J. Biol. Chem..

[85] Larson A.J., Symons J.D., Jalili T. (2012). Therapeutic Potential of Quercetin to Decrease Blood Pressure: Review of Efficacy and Mechanisms. Adv. Nutr..

[86] Russo M., Spagnuolo C., Tedesco I., Bilotto S., Russo G.L. (2012). The flavonoid quercetin in disease prevention and therapy: Facts and fancies. Biochem. Pharmacol..

[87] Zhu W., Gao J. (2008). The use of botanical extracts as topical skin-lightening agents for the improvement of skin pigmentation disorders. J. Invest. Derm. Symp. P..

[88] de Arriba S.G., Naser B., Nolte K.-U. (2013). Risk Assessment of Free Hydroquinone Derived from *Arctostaphylos Uva-ursi folium* Herbal Preparations. Int. J. Toxicol..

[89] Geetha R.V., Roy A., Lakshmi T. (2011). Nature’s weapon against urinary tract infections. Int. J. Drug Dev. Res..

[90] Halberstein R.A., Atta-ur-Rahman (2012). Botanical medicines for diuresis: Cross-cultural comparisons. Studies in Natural Products Chemistry.

[91] Tai A., Ohno A., Ito H. (2016). Isolation and Characterization of the 2,2′-Azinobis(3-ethylbenzothiazoline-6-sulfonic acid) (ABTS) Radical Cation-Scavenging Reaction Products of Arbutin. J. Agric. Food Chem..

[92] Bang S.H., Han S.J., Kim D.H. (2008). Hydrolysis of arbutin to hydroquinone by human skin bacteria and its effect on antioxidant activity. J. Cosmet. Dermatol..

[93] Pavlović R.D., Lakušić B., Došlov-Kokoruš Z., Kovačević N. (2009). Arbutin content and antioxidant activity of some Ericaceae species. Pharmazie.

[94] Takebayashi J., Ishii R., Chen J., Matsumoto T., Ishimi Y., Tai A. (2010). Reassessment of antioxidant activity of arbutin: Multifaceted evaluation using five antioxidant assay systems. Free Radic. Res..

[95] Li H., Jeong Y.M., Kim S.Y., Kim M.K., Kim D.S. (2011). Arbutin inhibits TCCSUP human bladder cancer cell proliferation via up-regulation of p21. Pharmazie.

[96] Cheng S.L., Liu R.H., Sheu J.N., Chen S.T., Sinchaikul S., Tsay G.J. (2007). Toxicogenomics of A375 human malignant melanoma cells treated with arbutin. J. Biomed. Sci..

[97] Lee H.J., Kim K.W. (2012). Anti-inflammatory effects of arbutin in lipopolysaccharide-stimulated BV2 microglial cells. Inflamm. Res..

[98] Taha M.M.E., Salga M.S., Ali H.M., Abdulla M.A., Abdelwahab S.I., Hadi A.H.A. (2012). Gastroprotective activities of Turnera diffusa Willd. ex Schult. revisited: Role of arbutin. J. Ethnopharmacol..

[99] Heaney R.P. (2001). Factors Influencing the Measurement of Bioavailability, Taking Calcium as a Model. J. Nutr..

[100] Carbonell-Capella J.M., Buniowska M., Barba F.J., Esteve M.J., Frígola A. (2014). Analytical methods for determining bioavailability and bioaccessibility of bioactive compounds from fruits and vegetables: A review. Compr. Rev. Food Sci. Food Saf..

[101] Richard W., Caballero B., Prentice A., Allen L. (2005). Bioavailability: Definition, general aspects and fortificants. Encyclopedia of Human Nutrition.

[102] Călinoiu L.F., Vodnar D.C. (2018). Whole Grains and Phenolic Acids: A Review on Bioactivity, Functionality, Health Benefits and Bioavailability. Nutrients.

[103] Lafay S., Gil-Izquierdo A. (2008). Bioavailability of phenolic acids. Phytochem. Rev..

[104] Olthof M.R., Hollman P.C., Zock P.L., Katan M.B. (2001). Consumption of high doses of chlorogenic acid, present in coffee, or of black tea increases plasma total homocysteine concentrations in humans. Am. J. Clin. Nutr..

[105] Falcone Ferreyra M.L., Rius S.P., Casati P. (2012). Flavonoids: Biosynthesis, biological functions, and biotechnological applications. Front. Plant. Sci..

