# Supplementary material for: Phenolic Compounds from Five Ericaceae Species Leaves and Their Related Bioavailability and Health Benefits
Source: Molecules. 2019 May 29;24(11):2046. doi: 10.3390/molecules24112046 (PMC6600139; doi:10.3390/molecules24112046)
Supplement: Supplementary file 1 [file molecules-24-02046-s001.pdf]

Phenolic compounds from five Ericaceae species leaves and their related bioavailability and several health benefits

Bianca Eugenia Ștefănescu[1,2], Katalin Szabo[2,*], Andrei Mocan[3,1] and Gianina Crişan[1,*]

[1]Department of Pharmaceutical Botany, "Iuliu Hațieganu" University of Medicine and Pharmacy, 23, Ghe. Marinescu Street, 400337Cluj-Napoca, Romania
[2]Institute of Life Sciences, University of Agricultural Sciences and Veterinary Medicine, Cluj-Napoca, CaleaMănăştur 3-5, 400372 Cluj-Napoca, Romania
[3]Laboratory of Chromatography, Institute of Advanced Horticulture Research of Transylvania, University of Agricultural Sciences and Veterinary Medicine, 400372 Cluj-Napoca, Romania

The phenolic compounds present in bilberry, lingonberry, bog bilberry, blueberry and bearberry leaves

	Compound name	BIL*	LIL*	BBL*	BLL*	BEL*
Antocyanins(only in the red leaves)	cyanidin-3-*O*-glucoside	d	nd	d	d	nd
	cyanidin-3-*O*-glucuronide	nd	nd	d	d	nd
	cyanidin-3-*O*-arabinoside	d	nd	d	d	nd
	cyanidin-3-*O*-galactoside	d	nd	nd	d	nd
Arbutin derivatives	Arbutin	nd	d	nd	nd	d
	2-*O*-caffeoylarbutin	nd	d	nd	nd	nd
	caffeoyl acetyl arbutin	nd	d	nd	nd	nd
	p-coumaroylarbutin	nd	d	nd	nd	nd
	p-coumaroyl acetyl arbutin	nd	d	nd	nd	nd
Catechins	Catechin	d	d	nd	nd	d
	epicatechin	d	d	nd	nd	d
	gallocatechin	d	nd	nd	nd	nd
	epigallocatechin	d	nd	nd	nd	d
Cinchonains	cinchonain I	d	d	d	d	nd
	cinchonain II	d	d	nd	nd	nd

Flavonols	Quercetin	d	d	nd	d	d
	quercetin-3-glucuronide	d	nd	nd	nd	nd
	quercetin-3-*O*-galactoside	d	d	d	d	nd
	quercetin-3-*O*-glucoside	d	d	d	d	d
	quercetin-3-*O*-rutinoside(rutin)	nd	d	nd	d	nd
	quercetin-3-*O*-xyloside	nd	d	nd	d	nd
	quercetin-3-*O*-arabinoside	d	d	nd	d	nd
	quercetin-3-*O*-arabinofuranoside	nd	d	nd	nd	nd
	quercetin-3-*O*-rhamnoside	d	d	d	nd	nd
	quercetin-3-*O*-(4″-HMG)-	d	d	nd	nd	nd
	rhamnoside	d	d	nd	nd	nd
	quercetin-3-(6″-acetyl) glucoside	nd	nd	nd	d	d
	kaempferol	d	nd	nd	nd	nd
	kaempferol-3-glucuronide	nd	d	nd	nd	nd
	kaempferol-(HMG)-rhamnoside	d	d	nd	nd	nd
	kaempferol-3-*O*-rhamnoside	d	d	nd	d	nd
	kaempferol-3-*O*-glucoside	nd	nd	d	nd	nd
	kaempferol-3-*O*-arabinoside	nd	nd	d	nd	nd
	kaempferol-feruloyl-	nd	nd	d	nd	nd
	acetylglucoside	d	nd	d	nd	d
	kaempferolcoumaroylglucoside	d	d	nd	d	d
	isorhamnetin 3-*O*-arabinoside					
	myricetin derivatives					
Iridoids	coumaroyliridoid isomers	d	d	d	nd	nd
Phenolic acids and derivatives	caffeic acid	d	d	nd	d	d
	p-coumaric acid	d	d	nd	d	nd
	caffeoylshikimic acid	d	d	nd	nd	d
	5-caffeoylquinic acid	d	d	d	d	nd
	4-caffeoylquinic acid	d	d	d	nd	nd
	3-caffeoylquinic acid	nd	d	nd	d	d
	dicaffeoylquinic acid	d	nd	nd	d	d
	caffeoyl hexose hydroxyphenol	nd	d	nd	nd	d
	5-*p*-coumaroylquinic acid	d	d	d	nd	nd
	4-*p*-coumaroylquinic acid	d	d	d	nd	nd
	3-*p*-coumaroylquinic acid	d	d	d	nd	nd
	coumaroyl hexose hydroxyphenol	nd	d	nd	nd	nd
	p-coumaroyldiacetylhexosides	d	nd	nd	nd	nd
	p-coumaroyltriacetylhexosides,	d	nd	nd	nd	nd
	p-coumaroylmalonylhexoside	d	nd	nd	nd	nd

	p-coumaroylmalonyldihexoside	d	nd	nd	nd	nd
	p-coumaroylmonotropein	d	d	nd	nd	nd
	feruloylquinic acid	d	d	d	nd	nd
	gallic acid derivative	d	nd	d	nd	d
	sinapic acid hexoside	nd	d	nd	nd	d
Proanthocyanidins	proanthocyanidin dimer type A	nd	d	nd	nd	nd
	proanthocyanidin trimer type A	d	d	nd	d	nd
	proanthocyanidin dimer type B	d	d	d	nd	nd
	proanthocyanidin trimer typeB	d	d	nd	d	nd
	proanthocyanidin tetramer type B	d	d	nd	nd	nd

*BIL-bilberry leaves; LIL-lingonberry leaves; BBL-bog bilberry leaves; BLL-blueberry leaves; BEL-bearberry leaves; d-detected; nd-not detected
